# Supplementary material for: Data-driven network alignment
Source: PLoS One. 2020 Jul 2;15(7):e0234978. doi: 10.1371/journal.pone.0234978 (PMC7331999; doi:10.1371/journal.pone.0234978)
Supplement: S3 Fig — Distribution of topological similarity (IsoRank) between node pairs of a (a,b,c) geometric and (d,e,f) scale-free network and their (a,d) 0%, (b,e) 25% randomly perturbed, and (c,f) 50% randomly perturbed counterparts. We show three lines representing the distribution of topological similarity for matching, i.e., functionally related, node pairs (blue), for non-matching, i.e., functionally unrelated, node pairs (red), and for 10 random samples of the same size as the set of matching pairs, averaged (purple). (PDF) [file pone.0234978.s003.pdf]

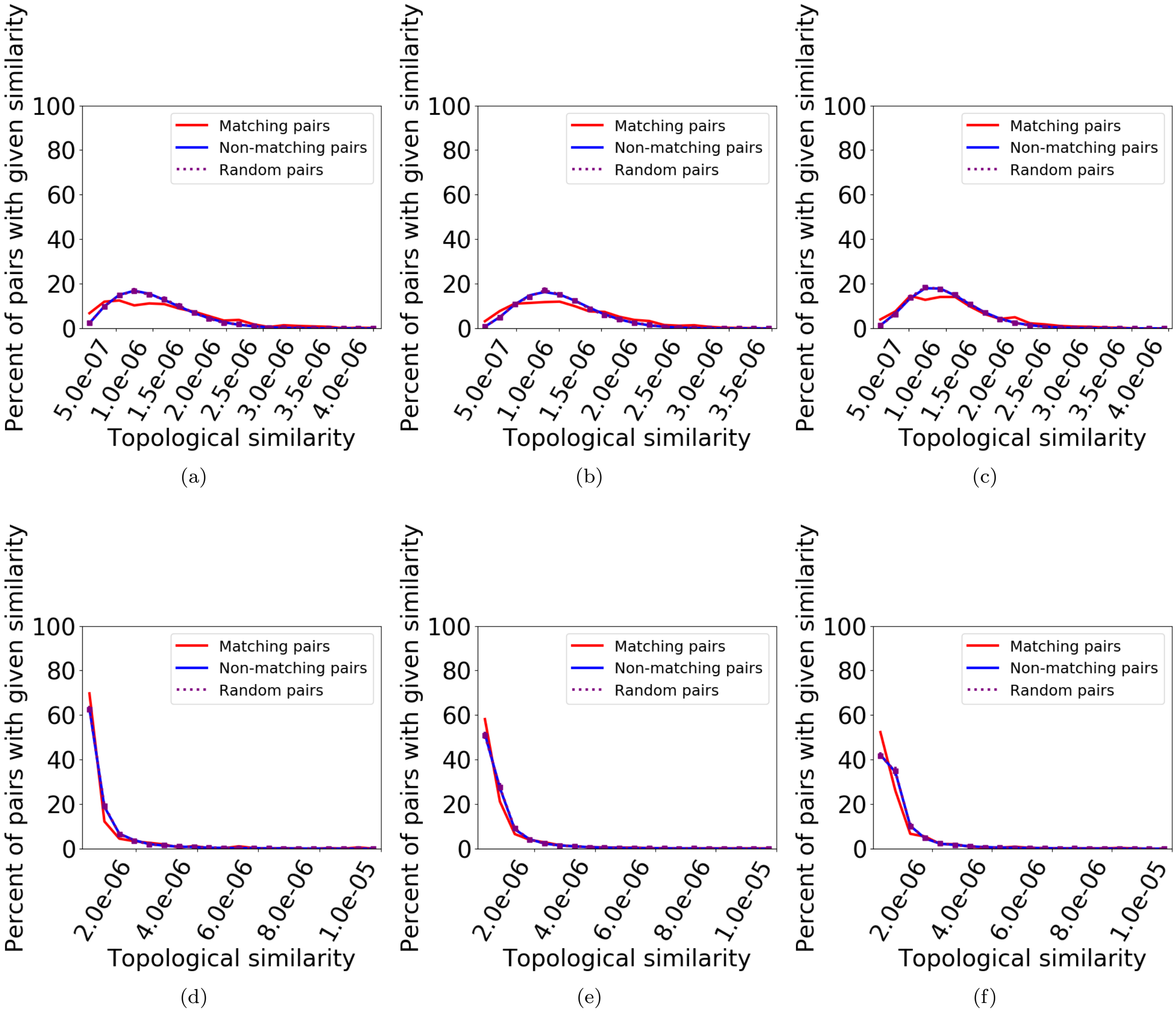

Supplementary Figure S3: Distribution of topological similarity (IsoRank) between node pairs of a **(a,b,c)** geometric and **(d,e,f)** scale-free network and their **(a,d)** 0%, **(b,e)** 25% noisy, and **(c,f)** 50% noisy counterparts. We show three lines representing the distribution of topological similarity for matching, i.e., functionally related, node pairs (blue), for non-matching, i.e., functionally unrelated, node pairs (red), and for 10 random samples of the same size as the set of matching pairs, averaged (purple).
