# Supplementary material for: Data-driven network alignment
Source: PLoS One. 2020 Jul 2;15(7):e0234978. doi: 10.1371/journal.pone.0234978 (PMC7331999; doi:10.1371/journal.pone.0234978)
Supplement: S4 Fig — Average (a,b) prediction accuracy and (c,d) AUROC of 10-fold cross-validation for (a,c) geometric and (b,d) scale-free networks. (PDF) [file pone.0234978.s004.pdf]

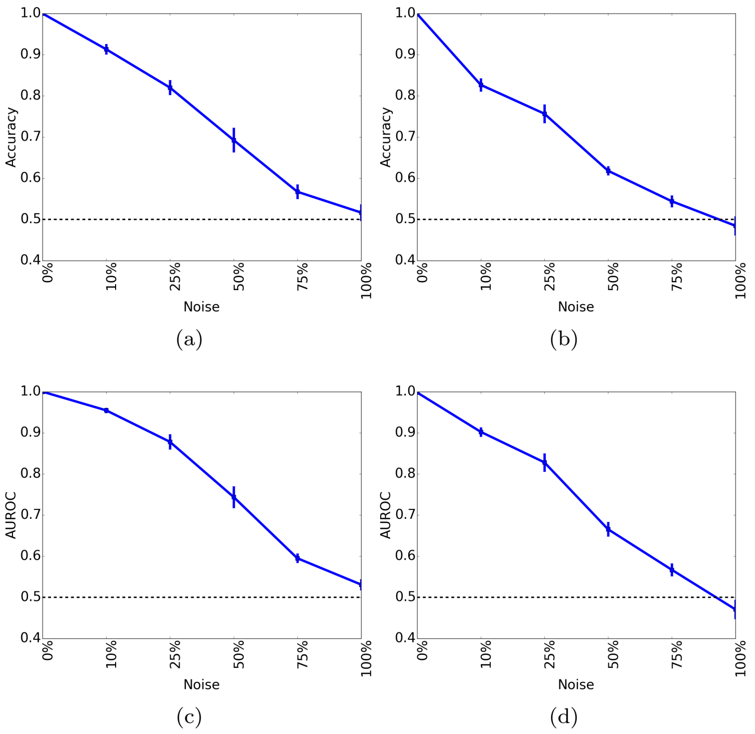

Supplementary Figure S4: Average **(a,b)** prediction accuracy and **(c,d)** AUROC of 10-fold cross validation for **(a,c)** geometric and **(b,d)** scale-free networks.
