# Supplementary material for: Data-driven network alignment
Source: PLoS One. 2020 Jul 2;15(7):e0234978. doi: 10.1371/journal.pone.0234978 (PMC7331999; doi:10.1371/journal.pone.0234978)
Supplement: S5 Fig — Average (a,b) prediction accuracy and (c,d) AUROC of percent training tests for (a,c) geometric and (b,d) scale-free networks. (PDF) [file pone.0234978.s005.pdf]

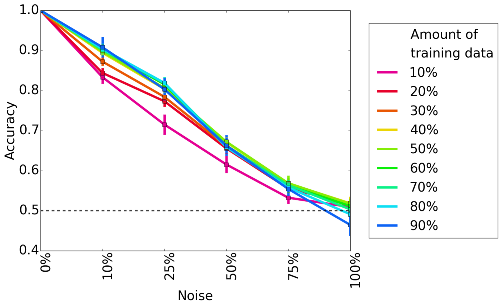

(a)

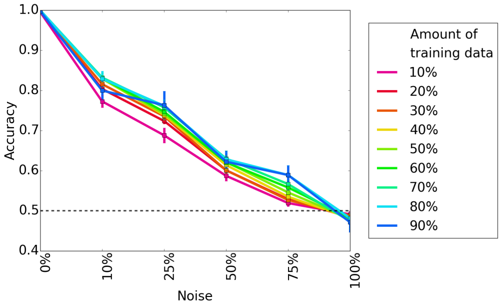

(b)

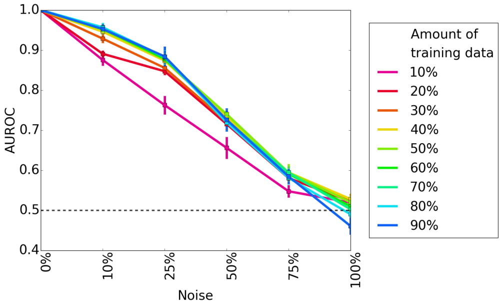

(c)

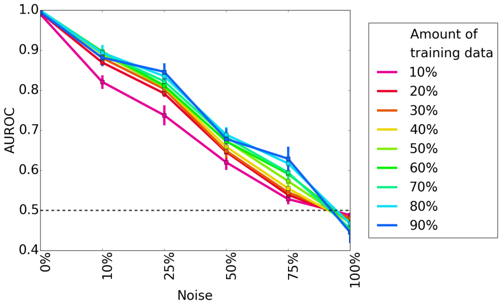

(d)

Supplementary Figure S5: Average **(a,b)** prediction accuracy and **(c,d)** AUROC of percent training tests for **(a,c)** geometric and **(b,d)** scale-free networks.
