# Supplementary material for: Data-driven network alignment
Source: PLoS One. 2020 Jul 2;15(7):e0234978. doi: 10.1371/journal.pone.0234978 (PMC7331999; doi:10.1371/journal.pone.0234978)
Supplement: S6 Fig — Average (a) prediction accuracy and (b) AUROC of 10-fold cross-validation for real-world networks. (PDF) [file pone.0234978.s006.pdf]

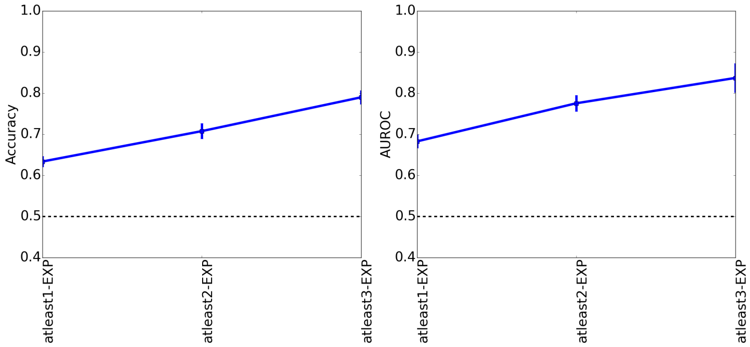

(a) (b)

Supplementary Figure S6: Average **(a)** prediction accuracy and **(b)** AUROC of 10-fold cross validation for real-world networks.
