# Supplementary material for: Data-driven network alignment
Source: PLoS One. 2020 Jul 2;15(7):e0234978. doi: 10.1371/journal.pone.0234978 (PMC7331999; doi:10.1371/journal.pone.0234978)
Supplement: S7 Fig — Average (a) prediction accuracy and (b) AUROC of percent training tests for real-world networks. (PDF) [file pone.0234978.s007.pdf]

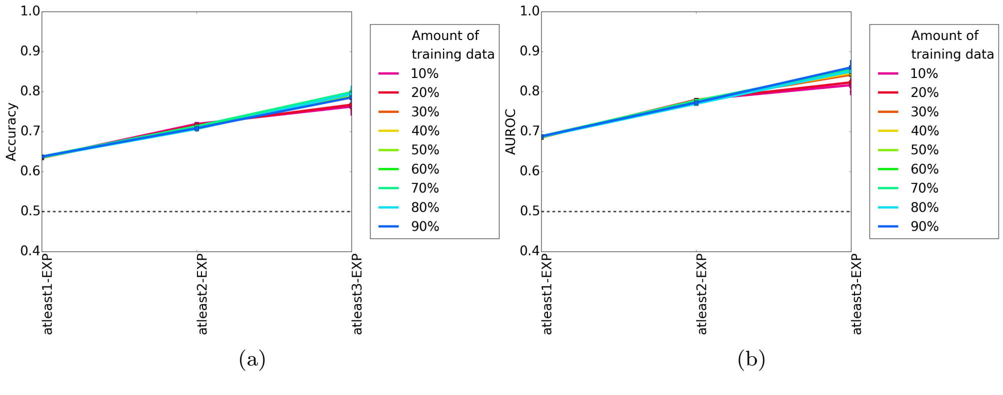

Supplementary Figure S7: Average **(a)** prediction accuracy and **(b)** AUROC of percent training tests for real-world networks.
