# Supplementary material for: Data-driven network alignment
Source: PLoS One. 2020 Jul 2;15(7):e0234978. doi: 10.1371/journal.pone.0234978 (PMC7331999; doi:10.1371/journal.pone.0234978)
Supplement: S8 Fig — Comparison of different TARA evaluation tests in the task of protein function prediction, for GO term rarity thresholds (a, d, g) ALL, (b, e) 50, and (c, f) 25 using ground truth datasets (a, b, c) atleast1-EXP, (d, e, f) atleast2-EXP, and (g) atleast3-EXP. Different percent training tests, specifically 10, 50, and 90, are compared within each panel. The alignment size (i.e., the number of aligned yeast-protein pairs) and number of functional predictions (i.e., predicted protein-GO term associations) made by each method, averaged over the 10 instances we perform for each test, are shown on the top. For example, the alignment for TARA-90 in panel (a) contains 27,155 aligned yeast-human protein pairs, and predicts 91,618 protein-GO term associations. Raw precision, recall, and F-score values are color-coded inside each panel. (PDF) [file pone.0234978.s008.pdf]

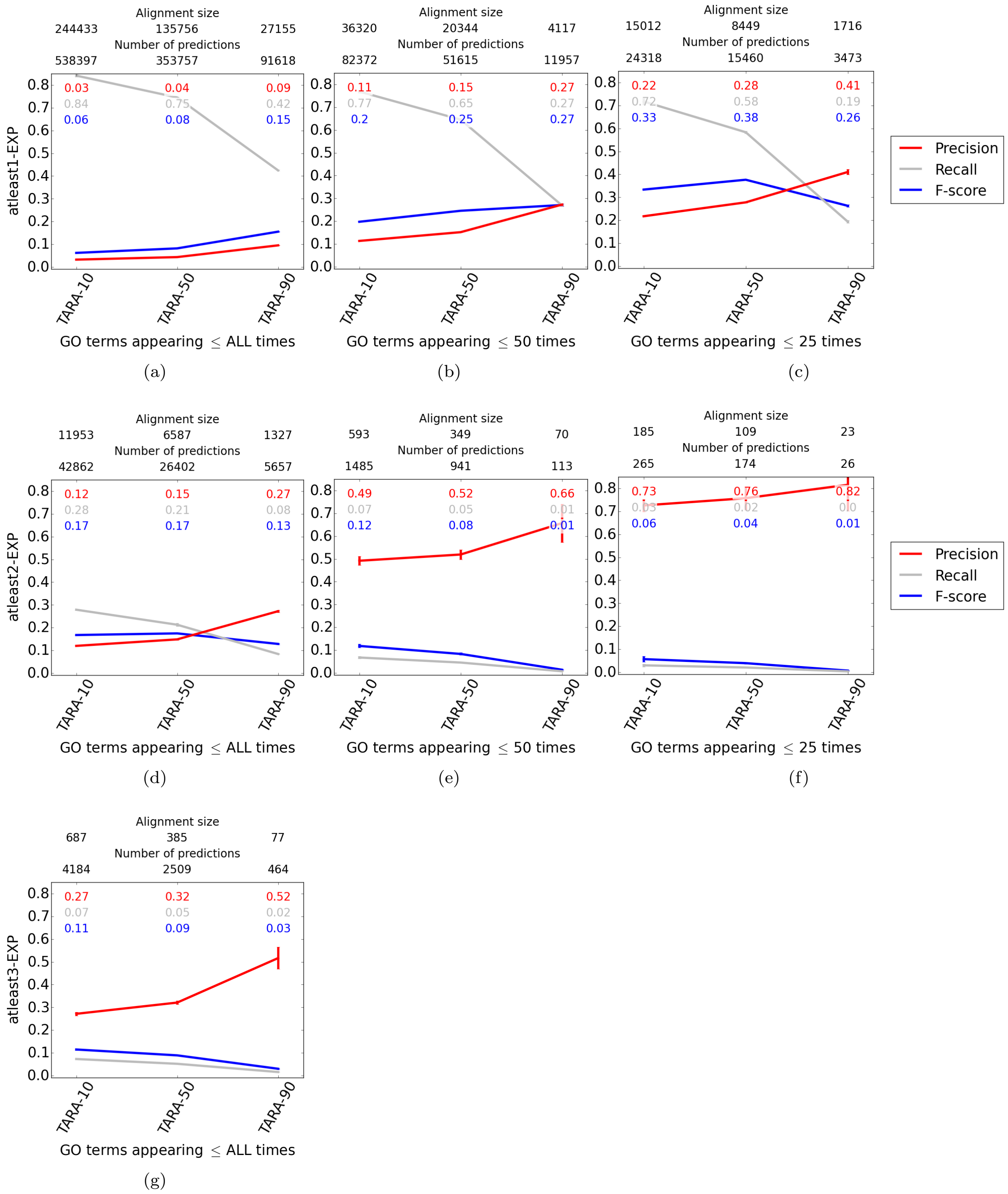


Supplementary Figure S8: Comparison of different TARA evaluation tests in the task of protein function prediction, for GO term rarity thresholds **(a, d, g)** ALL, **(b, e)** 50, and **(c, f)** 25 using ground truth datasets **(a, b, c)** atleast1-EXP, **(d, e, f)** atleast2-EXP, and **(g)** atleast3-EXP. Different percent training tests, specifically 10, 50, and 90, are compared within each panel. The alignment size (i.e., the number of aligned yeast-protein pairs) and number of functional predictions (i.e., predicted protein-GO term associations) made by each method, averaged over the 10 instances we perform for each test, are shown on the top. For example, the alignment for TARA-90 in **(a)** contains 27,155 aligned yeast-human protein pairs, and predicts 91,618 protein-GO term associations. Raw precision, recall, and F-score values are color-coded inside each panel.
