# Supplementary material for: Data-driven network alignment
Source: PLoS One. 2020 Jul 2;15(7):e0234978. doi: 10.1371/journal.pone.0234978 (PMC7331999; doi:10.1371/journal.pone.0234978)
Supplement: S10 Fig — Overlap of the functional predictions made by TARA and PrimAlign for GO term rarity thresholds (a, d, g) ALL, (b, e) 50, and (c, f) 25 using ground truth datasets (a, b, c) atleast1-EXP, (d, e, f) atleast2-EXP, and (g) atleast3-EXP. Percentages are out of the total number of unique predictions made by both methods combined. (PDF) [file pone.0234978.s010.pdf]

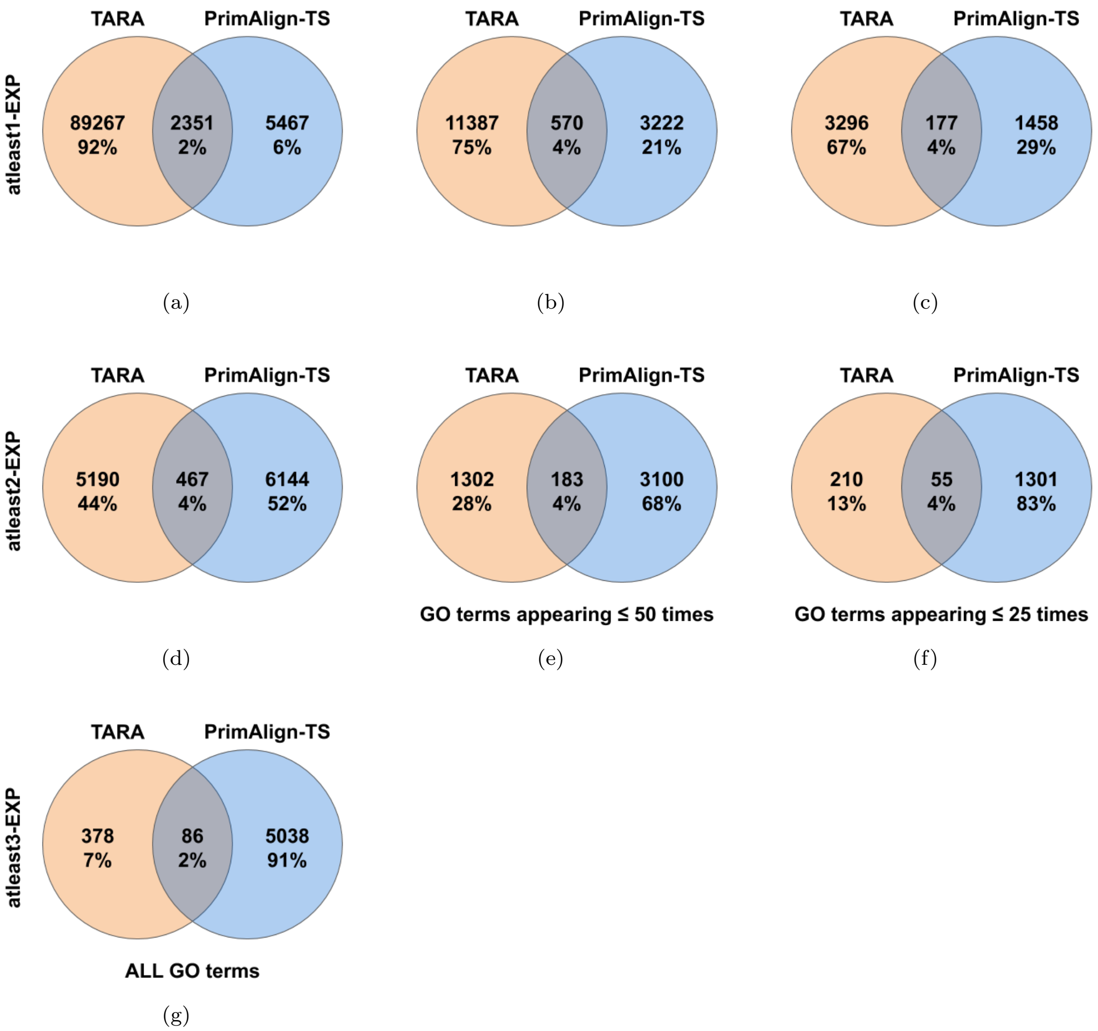

Supplementary Figure S10: Overlap of the functional predictions made by TARA and PrimAlign for GO term rarity thresholds **(a, d, g)** ALL, **(b, e)** 50, and **(c, f)** 25 using ground truth datasets **(a, b, c)** atleast1-EXP, **(d, e, f)** atleast2-EXP, and **(g)** atleast3-EXP. Percentages are out of the total number of unique predictions made by both methods combined.
