# Supplementary material for: Data-driven network alignment
Source: PLoS One. 2020 Jul 2;15(7):e0234978. doi: 10.1371/journal.pone.0234978 (PMC7331999; doi:10.1371/journal.pone.0234978)
Supplement: S11 Fig — Comparison of TARA on the 2017 versus 2020 networks for rarity thresholds (a, d, g) ALL, (b, e) 50, and (c, f) 25 using ground truth datasets (a, b, c) atleast1-EXP, (d, e, f) atleast2-EXP, and (g) atleast3-EXP in the task of protein function prediction. The alignment size (i.e., the number of aligned yeast-protein pairs) and number of functional predictions (i.e., predicted protein-GO term associations) made by each method. For example, the alignment for TARA-2017 in panel (a) contains 27,155 aligned yeast-human protein pairs, and predicts 91,618 protein-GO term associations. Raw precision, recall, and F-score values are color-coded inside each panel. (PDF) [file pone.0234978.s011.pdf]

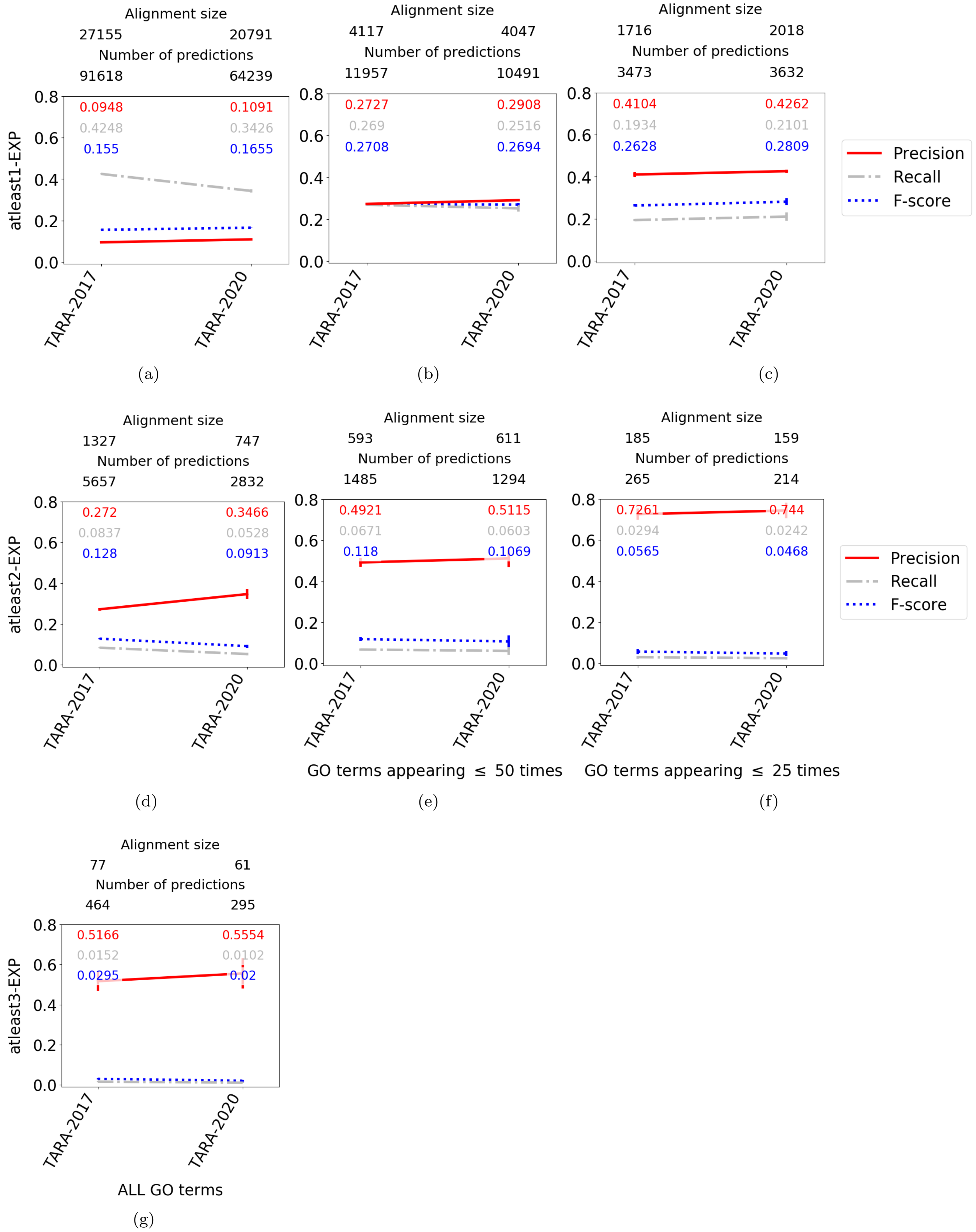

Supplementary Figure S11: Comparison of TARA on the 2017 versus 2020 networks for rarity thresholds **(a, d, g)** ALL, **(b, e)** 50, and **(c, f)** 25 using ground truth datasets **(a, b, c)** atleast1-EXP, **(d, e, f)** atleast2-EXP, and **(g)** atleast3-EXP in the task of protein function prediction. The alignment size (i.e., the number of aligned yeast-protein pairs) and number of functional predictions (i.e., predicted protein-GO term associations) made by each method. For example, the alignment for TARA-2017 in panel **(a)** contains 27,155 aligned yeast-human protein pairs, and predicts 91,618 protein-GO term associations. Raw precision, recall, and F-score values are color-coded inside each panel.
