# Supplementary material for: Data-driven network alignment
Source: PLoS One. 2020 Jul 2;15(7):e0234978. doi: 10.1371/journal.pone.0234978 (PMC7331999; doi:10.1371/journal.pone.0234978)
Supplement: S1 Table — (PDF) [file pone.0234978.s012.pdf]

www.nd.edu/~cone/TARA/alignment-prediction-stats-tara-vs-primalign-ts.xlsx

Supplementary Table S1: Detailed statistics regarding predictions made by TARA and PrimAlign-TS.
